# Post‐hematopoietic stem cell transplantation relapse: Role of checkpoint inhibitors

**DOI:** 10.1002/hsr2.536

**Published:** 2022-03-08

**Authors:** Elham Roshandel, Farzaneh Tavakoli, Sayeh Parkhideh, Sedigheh Sadat Akhlaghi, Maria Tavakoli Ardakani, Masoud Soleimani

**Affiliations:** ^1^ Hematopoietic Stem Cell Research Center Shahid Beheshti University of Medical Sciences Tehran Iran; ^2^ Department of Internal Medicine, School of Medicine, Ayatollah Taleghani Hospital Shahid Beheshti University of Medical Sciences Tehran Iran; ^3^ Department of Clinical Pharmacy, School of Pharmacy Shahid Beheshti University of Medical Sciences Tehran Iran

**Keywords:** checkpoint blocker, hematopoietic stem cell transplantation, immune checkpoint, relapse

## Abstract

**Background and Aims:**

Despite the revolutionary effects of hematopoietic stem cell transplantation (HSCT) in treating hematological malignancies, post‐HSCT relapse is considered a critical concern of clinicians. Residual malignant cells employ many mechanisms to evade immune surveillance and survive to cause relapse after transplantation. One of the immune‐frustrating mechanisms through which malignant cells can compromise the antitumor effects is misusing the self‐limiting system of immune response by overexpressing inhibitory molecules to interact with the immune cells, leading them to so‐called “exhausted” and ineffective. Introduction of these molecules, known as immune checkpoints, and blocking them was a prodigious step to decrease the relapses.

**Methods:**

Using keywords *nivolumab*, *pembrolizumab*, and *ipilimumab*, we investigated the literature to figure out the role of the immune checkpoints in the HSCT setting. Studies in which these agents were administrated for relapse after transplantation were reviewed. Factors such as the interval from the transplant to relapse, previous treatment history, adverse events, and the patients’ outcome were extracted.

**Results:**

Here we provided a mini‐review discussing the experiences of three immune checkpoints, including nivolumab, pembrolizumab, and ipilimumab, as well as the pros and cons of using their blockers in relapse control after HSCT. In conclusion, it seems that CI therapy seems effective for this population. Future investigations may provide detailed outlook of this curative options.

## 1 INTRODUCTION

Hematopoietic stem cell transplantation (HSCT) has been proposed as a therapeutic strategy for hematological malignancies, although subsequent complications still attenuate favorable outcomes.^1^ With the advent of non‐myeloablative reduced‐intensity conditioning (RIC) and allogeneic HSCT (allo‐HSCT), the spectrum of patients’ candidates for transplantation has been widened. However, infection, graft‐versus‐host disease (GVHD), and relapse are the main obstacles that decrease the success of allo‐HSCT. With a 1‐year survival below 20%, relapse is the most common cause of treatment failure after HSCT.^2^ Post‐transplantation strategies for reducing relapses, such as donor lymphocyte infusion (DLI) and donor‐derived T/natural killer (NK) cell infusion, could not succeed as much as expected due to the potential complications and risks.^3^ DLI is often limited to chronic myeloid leukemia (CML) patients due to the increasing high risk of GVHD in others^4^; NK cell therapy is still hesitating concerning difficulties in its expansion and standardization protocol. Therefore, a thoughtful choice of alternative treatment for post‐HSCT relapse deserves more investigations.^4^

Survived leukemic stem cells (LSCs) from conditioning regimens and poor immune reconstitution are responsible for relapse after HSCT.^5^ There is evidence that niche interactions between bone marrow stroma and LSCs provide protective properties against chemotherapy.^6^ Also, one of the reasons for the resistance of LSCs is their dormant status.^7^ Releasing exosomes and microvesicles, LSCs alter the healthy niche to leukemic‐supportive microenvironment.^8^ Thanks to the favorable effect of graft‐versus‐leukemia (GVL), the relapse rate is lower in allo‐HSCT than autologous HSCT (auto‐HSCT). Therefore, relapse may be a hint of the failure of GVL. In other words, mechanisms that increase the GVL effect can also increase GVHD, which explains why less relapse occurs in patients with a history of mild GVHD.

Immune cells have a substantial role in relapse control. Reduced dendritic cells (DCs) have been associated with relapse after HSCT.^9^ Although regulatory T cells (T regs) effectively prevent GVHD, they may prohibit GVL.^10^ Consistent with this, it has been demonstrated in a study that a higher dose of CD34+ cells correlates with a higher rate of relapse, which proposed that it could be due to more malignant cells and T regs, decreasing antileukemic effects.^11^ Because of a higher percentage of T cells in peripheral blood HSCT, the relapse rate is lower at the expense of a higher incidence of GVHD.^12^ NK cells can also be considered as another critical relapse‐inhibiting agent because relapse in high‐risk acute myeloid leukemia (AML) patients transplanted by killer immunoglobulin‐like receptor (KIR)‐ligand mismatch was 0%.^13^ Similarly, a study conducted by Willem et al.^14^ demonstrated that KIR/human leukocyte antigen (HLA) incompatibility has favorable effects in reducing relapse.

Sometimes, despite the sufficient number, lymphocytes are not functional due to the inhibitory interaction between leukemic and immune cells. Impaired function of B and T lymphocytes is associated with relapse after HSCT. In patients who relapsed after HSCT, bone marrow‐infiltrating CD8+ T cells have a unique immunophenotype signature called “exhausted,” which expresses a high level of programmed cell death protein 1 (PD1), cytotoxic T‐lymphocyte‐associated protein 4 (CTLA4), and/or other immune‐checkpoints (CI). The presence of exhausted CD8+ T cells and the immunosuppressive state of the niche in relapsed myeloma reveal the potential of immunotherapy in preventing disease recurrence.^15^ Regarding the checkpoint's role in various malignancies, checkpoint inhibitors (CIs) have been introduced for disease treatment and management. Evaluation of CIs in some malignancies displayed satisfactory results, especially in melanoma and lung cancers. In hematological disorders, there are growing studies aiming at the effect of CIs in relapse management. Here we reviewed the role of CIs in the HSCT setting.

## 2 PD1 BLOCKADE IN POST‐HSCT RELAPSE

Recently, the application of PD1 inhibitors in hematological malignancies has been strikingly growing. Since the Hodgkin lymphoma (HL) microenvironment demonstrates unique features, it is potentiated to get the advantage of CIs. There are a few Red‐Sternberg cells and numerous ineffective immune cells in the lymphoma niche.^16^ Therefore, most of the investigations are focused on refractory/relapsed HD (R/R HD).

It has been reported that the expression of inhibitory molecules, such as PD1 on the T‐cell surface is associated with relapse after HSCT, independent of GVHD. The predictive value of PD1‐high immune cells for leukemia relapse post‐HSCT has also been suggested.^15^ Experimental evidence supporting the role of the PD1/PD1 ligand (PDL1) pathway in post HSCT relapse is conducted by Norde et al. study, in which they observed high expression of PDL1 in CD34+ leukemic cells in relapsed patients. They reported that leukemic cells that do not express costimulatory molecules such as CD80 and CD86 make the immune system unqualified for fighting them.^17^ Accordingly, targeting the PD1 molecular pathway by immune CIs can break tumor immune tolerance and preclude the cytotoxic T cells’ deactivation.^15^, ^18^ Response induction of anti‐PD1 therapy seems to be applied through several different mechanisms, among which the changes in transcription and translation of immune checkpoints associated genes have been demonstrated in tumor infiltrated lymphocytes.^19^, ^20^ Another evidence for the significance of PD1 inhibition timing, deduced from Koestner et al. experience,^21^ in which attenuated GVL is associated with increased expression of PD1 in the allogeneic recipients. They reported that delayed adoptive transfer needs PD1/PDL1 inhibition to promote GVL effects. GVL reviving could occur by PDL1 inhibition without causing GVHD. In contrast, coadministration of PDL1 blockers with the early adoptive transfer may induce fatal GVHD, which is the main concern in using these drugs.^22^

Given the fact that the antitumor effects of GVL have pertained to the donor T cells, it has been concluded that PD‐1 blockade could induce remission in a patient unresponsive to previous DLI therapy.^23^ On the other hand, a recent study suggested that although CI therapy plus DLI reduce post‐HSCT relapse, it also induces GVHD potential in many patients, highlighting the importance of patient selection and appropriate timing of the CI administration.^24^ Thus, CI therapy alone may have more acceptable results than combining with DLI in some cases. Two prominent PD1 inhibitors, including nivolumab and pembrolizumab, have been investigated in transplantation studies. Here, we reviewed the literature on these CIs.

## 3 NIVOLUMAB AND STEM CELL TRANSPLANTATION

Nivolumab, a humanized IgG4 kappa monoclonal antibody, has been approved for relapse treatment after auto‐SCT since 2016.^25^ There are many promising results about using nivolumab in post‐HSCT relapse. One prominent example was a patient with Hodgkin disease who experienced chemotherapy and relapsed after auto‐HSCT. Using a PD1 blocker, 32 months after allo‐HSCT, he achieved complete remission (CR).^26^ Nivolumab induces remission in patients after auto‐HSCT and failure of brentuximab vedotin, an anti‐CD30 antibody.^27^ Nivolumab demonstrated its efficiency for HD patients who experienced relapse after allo‐SCT with a more acceptable risk−benefit ratio than other treatment options such as brentuximab vedotin and DLI. In this study, no GVHD was observed in patients without a history of acute GVHD.^28^

While most studies focused on the effect of this drug on post‐transplantation relapse, there are other studies administrating nivolumab at other time points. Manson et al. study revealed that prescribing anti‐PD1 before allo‐SCT may increase GVL; in this condition, SCT can be considered a consolidation treatment. Notably, all the patients in their study experienced GVHD.^29^ Similarly, another study on the 74 R/R HL suggested allo‐SCT as a suitable consolidation therapy.^30^ Sindel et al.^31^ demonstrated that pretransplantation administration of nivolumab resulted in a cytokine storm, which could be controlled by ascorbic acid. Also, a high risk of early immune toxicity was reported from a study conducted on 39 cases.^32^ As an effort to find other suitable timing of PD1 inhibitors, a 2017 study explained the beneficial effect of nivolumab plus brentuximab. It could pave the way for transplantation for R/R HL patients who are SCT‐ineligible.^33^

While most investigations have been done on R/R HD, several studies reported the experience of nivolumab therapy in different hematological disorders. A study conducted on AML patients using nivolumab as a first‐line treatment indicated that it does not increase the incidence of grade 3−4 GVHD.^34^ Wang et al. demonstrated the importance of the timing of nivolumab administration in AML patients as maintenance treatment; all cases showed immune‐related adverse events. The author suggested that high toxicity would observe when nivolumab began 7.8 months after allo‐SCT.^35^

One of the rare challenges after allogeneic transplantation is post‐transplantation lymphoproliferative disorders (PTLD), which result from disrupted immunological surveillance. The main factors that affect the occurrence of PTLD are the type of donor and the T‐cell depletion method. The rate of PTLD after allo‐HSCT has been reported as below: 4%−10% unrelated donor transplantation and 1%−3% matched related donor.^36^ While most previous studies have examined the impact of this drug on the recurrence of the primary disease, another group of investigators administrated PD1 inhibitors in transplantation lymphoproliferative disorders (PTLD). Nivolumab successfully induced immune response in a patient diagnosed with myelofibrosis and experienced HL‐like PTLD.^37^ Another successful treatment of PTLD with nivolumab was achieved by Wada et al.^38^ with the cost of liver toxicity. A similar indication of nivolumab was reported from a pediatric setting. An 11‐year‐old child diagnosed with primary CML demonstrated primary central nervous system PTLD. This girl was responsive to nivolumab after the failure of methotrexate and rituximab^39^ (Table 1).

**Table 1:** The studies of nivolumab after relapse of HSCT

Author	Year	Patients		Diagnosis	DLI	Outcome	Comment	Ref.
Ansell	2015	18 with previous auto‐SCT		HL	+	ORR of 87% and a rate of PFS of 86% at 24 weeks, Beneficial	Auto‐SCT, relapse	[40]
Dada	2016	1		HL	−	Major response	Previous treatment: auto‐SCT	[41]
Albring	2016	3		AML	2 cases	Three different responses: CR, disease stabilization, failed to respond	Allo‐SCT, relapse	[42]
Falchi	2016	2		HL	−	High rate of complete responses	Auto‐SCT, relapse,	[43]
Shad	2016	1		HL	−	No GVHD, tolerable	Auto‐SCT, haploidentic allo‐SCT, relapse	[44]
Klobuch	2017	2		HL	+	1 case GVHD	Allo‐SCT, relapse‐CI	[45]
McDuffee	2017	1		HL	+	Alive with GVHD	Allo‐SCT, relapse	[46]
Bekoz	2017	57		HL	−	2 GVHD, One expired	55 previous auto‐SCT, 16 previous allo‐SCT	[47]
Haverkos	2017	31		29 HL/2 lymphoma	5 cases	8 cases died of GVHD (high rate of GVHD)	Allo‐SCT, relapse	[48]
Jiménez‐Ubieto	2017	1		HL	−	Fatal GVHD, longer interval between nivolumab and allo‐SCT	Allo‐SCT, relapse	[49]
Chan	2017	2		ALL	1 case	One case: hepatic cGVHD, overall beneficial	Allo‐SCT, relapse	[50]
Covut	2017	5 (3 received CI before allo‐SCT)		HL	−	Suitable alternative treatment	Auto‐SCT, allo‐SCT, relapse	[51]
Onizuka	2017	1		HL	+	Manageable GVHD	Auto‐SCT, allo‐SCT, relapse	[52]
Cheikh	2017	2		HL	+	One case died of GVHD	Auto‐SCT, allo‐SCT, relapse	[53]
Terziev	2018			Primary CNS lymphoma	−	Remission	3rd auto‐SCT, relapse	[54]
Ansell	2019	87		DLBCL		Beneficial after failure of auto‐SCT	Auto‐SCT, relapse	[55]
Forceville	2019	2		HL	−	CMR, radiotherapy in combination with nivolumab	Auo‐SCT, relapse,	[56]
Charles	2019	1		HL	−	Multi‑organ failure, died	Auto and allo‐SCT, CI, haplo‐allo‐SCT	[57]
Wong	2019	1		T‐AML	−	Refractory to nivolumab	UR‐ allo‐SCT, relapse	[58]
Lepik	2019	40		HL	−		Auto‐SCT relapse	[59]
Ogasawara	2019	1		HL	−	Refractory to nivolumab, expired of relapse, GVHD	Auto & haplo‐allo‐SCT, relapse	[60]
Kobayashi	2019	1		HL	−	CR, myositis	Auto‐SCT, Allo‐SCT, relapse	[61]
Quéro	2019	3		HL	−	In combination with radiotherapy, lung toxicity, CMR	Auto‐SCT, relapse	[62]
Chan	2020	6 (11 received pembrolizumab)		HL	−	ORR: 100%, PFS: 33 months, low dose was effective	17% auto‐SCT, 34% allo‐SCT	[63]
Sim	2020	1		ALL	−	CR with mild cGVHD		[64]

Abbreviations: ALL, acute lymphoblastic leukemia; allo‐SCT, allogeneic stem cell transplantation; AML, acute myeloid leukemia; auto‐SCT, autologous stem cell transplantation; cGVHD, chronic graft versus host disease; CI, check point inhibitor; CMR, complete metabolic response; CNS, central nervous system; CR, complete remission; DLBCL, diffuse large B‐cell lymphoma; GVHD, graft versus host disease; Haplo, haploidentical; HL, Hodgkin lymphoma; ORR, overall response rate; PFS, progression‐free survival; T‐AML, therapy‐related acute myeloid leukemia.

## 4 PEMBROLIZUMAB AND STEM CELL TRANSPLANTATION

### 4.1 Introduction and approval Hodgkin lymphoma

Another PD1 inhibitor agent is pembrolizumab which is a humanized IgG4 antibody. Pembrolizumab is approved for relapsed or refractory classical Hodgkin lymphoma in 2020. Clinical trials approved administration of 200 mg every 3 weeks is effective for relapsed Hodgkin lymphoma.^65^ The results of clinical trials led to the approval of this new agent.^66^ An analysis conducted by Armand et al. demonstrated that receiving pembrolizumab after SCT seems beneficial with a 73% of overall response rate (ORR), 59% partial remission, and 14% complete remission. They also could show that this medication induces upregulation of IFN‐gamma.^67^ In a report of two cases of HL after allo‐SCT, safe and successful treatment was obtained by achieving partial and complete remission.^68^ Chan et al. shared their results on using CI agents before auto‐SCT. Evaluation of pembrolizumab therapy in 5 R/R HL patients with a history of failure in different treatment strategies showed 100% ORR and almost no toxicity was observed.^69^ Strengthening the immune system is always likely to cause unwanted side effects, as some degree of autoimmunity, which can sometimes be a disaster for the patient. The first fatal GVHD after pembrolizumab treatment was reported in 2016. The patient died due to skin and lung chronic GVHD.^70^ Another evidence is a report of post‐pembrolizumab insulin‐dependent diabetes in a 12‐year‐old boy with a history of disease recurrence after auto‐SCT. Getting rid of lymphoma came at the cost of developing diabetes for this patient, highlighting the importance of cautiousness when applying CI agents in the pediatric setting.^71^ In this regard, the findings of a study that revealed two death out of three indicate the importance of dose optimizing in preventing complications.^72^

### 4.2 Pembrolizumab and  SCT in other leukemia/lymphoma

Like nivolumab, the number of studies on pembrolizumab in HL is much higher than in other blood disorders. Recently, various hematological diseases were added to the list of research on pembrolizumab's role in their treatment. Frigault et al. examined 29 diffuse large B‐cell lymphoma patients who received pembrolizumab to evaluate the effect of this CI as consolidation treatment after auto‐SCT. Since the 18‐month progression‐free survival was 59%, it can be concluded that the results are not very satisfactory.^68^ Promising results have been detected in the anaplastic large‐cell lymphoma patients. A study reported that a relapsed patient with the previous auto‐ and allo‐SCT was asymptomatic after receiving pembrolizumab. Though, it should be noted that she experienced hepatic GVHD.^73^ In the case of NK/T lymphoma, the patient who received pembrolizumab before manipulated haploidentical transplantation achieved an almost complete response.^74^

There are a couple of investigations on multiple myeloma patients. Bryant et al. represented a multiple myeloma patient who received pembrolizumab on +118 and developed severe neutropenia. Their results emphasize the importance of immune complications after the administration of this drug.^75^ Another study on multiple myeloma patients indicated that pembrolizumab and lenalidomide might have a positive effect at the beginning, on the day of +14, as maintenance treatment.^76^

Hsiao et al. follow upped two AML patients with relapse after allo‐SCT that received pembrolizumab. GVHD occurred in both patients, and one of them expired. Dried eyes are documented in both of them.^77^

A rapid and durable response was observed after pembrolizumab administration in a sezary syndrome patient who relapsed after allo‐SCT.^78^

## 5 CTLA4 BLOCKADE IN POST‐HSCT RELAPSE

Cytotoxic T‐lymphocyte antigen 4 (CTLA4) is an inhibitory receptor, expressed on activated memory T cells and regulatory T cells, that can bind to CD28 on immune cells and negatively regulate their excess activation and functions. Besides, CTLA4 can impair the interaction between CD28 and its costimulatory ligands (CD80 and CD86) by trans‐endocytosis of CD28. The effect of donor CTLA4 polymorphisms on the transplant outcome, overall survival, and relapse have been discussed in several studies.^79^, ^80^ In a very recent study on the pediatric population, polymorphism in donor CTLA4 was associated with a lower rate of relapse and no increase in GVHD incidence.^79^ CTLA4 targeting has been investigated in some HSCT conditions with controversial results. It has been shown that blocking CTLA4 in post‐transplantation relapse could activate T cells,^81^ raising the possibility of immune‐related adverse effects.

Ipilimumab, a fully humanized IgG1 monoclonal antibody against CTLA4, is the first and only FDA‐approved CTLA4 inhibitor for metastatic melanoma. Ipilimumab has been used for many hematological disorders after relapse. In recent years, ipilimumab has also been tried for some post‐SCT relapsed hematological disorders.^82^ Accordingly, Bashey et al. reported organ‐specific immune adverse events concurrent with tumor regression in 14% of cases. However, no acute or chronic GVHD was observed.^83^ Later, Davids et al.^84^ proposed the late administration of ipilimumab in post‐HSCT relapse for more beneficial responses. However, it should be noted that the absence of severe GVHD was one of the inclusion criteria for both of these two studies. Therefore, the previous complications of patients must be considered before patient selection for this treatment strategy. Another study has investigated ipilimumab in combination with lenalidomide for patients who relapsed after allogeneic and autologous HSCT. The response rate was higher than the expectations for allo‐HSCT patients, probably due to lenalidomide addition.^85^ Another evidence is extracted from a report by Gros et al. representing a young man with early relapse after haploidentical SCT administered with ipilimumab on Day +63. Progressive GVHD and death occurred 19 days after receiving CI.^86^ It can be concluded that clinicians should be cautious for ipilimumab administration after haplo‐transplantation. Supporting this, another report from CI administration in a myelofibrosis patient who relapsed after second haploidentical transplantation showed autoimmune hemolytic anemia as an adverse effect of ipilimumab.^87^

## 6 BTLA4 BLOCKADE IN POST‐HSCT RELAPSE

B and T lymphocyte attenuator (BTLA4) is an inhibitory receptor that belongs to the super immunoglobulin family and induces its suppression effect on T cells through interaction with a receptor called herpesvirus entry mediator (HVEM).^88^ HVEM, a member of the tumor necrosis factor receptor superfamily, is constitutively expressed on malignant cells in leukemia. Blocking this pathway is reasonable to prevent disease recurrence after HSCT. This interaction impaired the T‐cell response to tumor relapse. In some patients, BTLA blocking results in more noticeable effects than PD1 therapy.^88^

The association between BTLA and GVHD has been discussed in preclinical investigations and showed that, in the first days after HSCT, the anti‐BTLA antibody could control GVHD, maintain the GVL effect through deactivating donor T cells, and cause a relative increase in natural regulatory T cells.^89^ Contrarily, del Rio et al.^90^ suggested that blocking BTLA/HVEM pathway is insufficient to inhibit the infiltration and allo‐reaction of donor T cells to GVHD susceptible tissues. Figuring out the exact effect of blocking the BTLA pathway on GVL and GVHD needs more investigations and an extended follow‐up period.

## 7 COMPLICATIONS OF CI THERAPY IN HSCT

A meta‐analysis demonstrated rare fatal toxic effects due to CI therapy. The highest rate of complications was reported when PD1/PDL1 and CTLA4 inhibitors were administered together, which only occurred in 1.23% of patients. The most involved organs were the colon, liver, lungs, pituitary, thyroid, and skin.^91^ Kaloyannidis et al. reported that a patient who received nivolumab before transplantation showed de novo Psoriasis Vulgaris, which resolved after auto‐HSCT. It has been presumed that conditioning regimens (Melfalan) and auto‐HSCT could eliminate skin complications.^92^ A literature review by Ijaz et al.^4^ reported 14% acute and 9% chronic GVHD, following CI therapy post‐HSCT; 28% of post‐allo‐HSCT relapsed patients achieved complete remission.

Interestingly, it has been revealed that shorter interval between HSCT and CI administrations increases the risk of GVHD.^93^ Other factors influencing GVHD probability include GVHD prophylaxis regimen, donor source, previous history of GVHD, and concurrent immunosuppression with CI.^94^, ^95^

Further studies may shed light on the best time for starting CI therapy. There is also a lack of data regarding the appropriate dose of CI administration. Assessment of PDL1 expression on post relapse lesions by immunohistochemistry might be informative about choosing an appropriate dose of nivolumab. In the field of adoptive cell therapy, coadministration of CI agents such as anti‐PD1 and adoptive T/NK cell therapy raises the cytotoxicity of effector cells and improves the efficacy of procedures.^3^, ^4^ These results may light the interest in adding CI therapy to other treatment approaches.

## 8 CONCLUSION

Allogeneic transplantation is performed in patients who have already experienced one or multiple relapses before being candidates for transplantation. Therefore, they have limited therapeutic options following post‐HSCT relapse because treatment options such as auto‐HSCT and alternative chemotherapy have been tried for many patients. Based on previous studies, CI therapy could be a promising treatment for some relapsed patients. However, some tips should be addressed before CI prescription. (I) Administration of these medications for patients with a history of autoimmunity or severe GVHD may result in serious complications. (II) Expression assessment of the checkpoints and their ligands on malignant and immune cells seems to be necessary to achieve favorable results. (III) Type, dose, and time to start CI therapy could have a tremendous effect on the outcome. (IV) Combining two drugs for patients without favorable response after single CI therapy could be beneficial while considering the possible increase in adverse effects. Finally, leukemia/lymphoma may be weakened by HSCT, but they persist and can relapse. However, combining therapy may eliminate it entirely or make it so weak that no annoying symptoms could be observed.

## CONFLICTS OF INTEREST

The authors declare no conflicts of interest.

## AUTHOR CONTRIBUTIONS

*Contributed in concept, data collection*: Elham Roshandel. *Writing—original draft preparation*: Sayeh Parkhideh and Maria Tavakoli Ardakani. *Writing—review and editing*: Sedigheh Sadat Akhlaghi. *Conceptualization, investigation, interpreted and approved the article*: Elham Roshandel and Farzaneh Tavakoli. *Methodology and supervision*: Masoud Soleimani. All authors approved the final version of the manuscript.

## References

[1] Ardakani MT , Mehrpooya M , Mehdizadeh M , Beiraghi N , Hajifathali A , Kazemi MH . Sertraline treatment decreased the serum levels of interleukin‐6 and high‐sensitivity C‐reactive protein in hematopoietic stem cell transplantation patients with depression; a randomized double‐blind, placebo‐controlled clinical trial. Bone Marrow Transplant. 2019;55(4):830‐832. 31383995 10.1038/s41409-019-0623-0

[2] Bazarbachi A , Labopin M , Battipaglia G , et al. Sorafenib improves survival of FLT3‐mutated acute myeloid leukemia in relapse after allogeneic stem cell transplantation: a report of the EBMT Acute Leukemia Working Party. Haematologica. 2019;104(9):e398. 30792203 10.3324/haematol.2018.211615 PMC6717576

[3] Liu K , Chen Y , Zeng Y , et al. Coinfusion of mesenchymal stromal cells facilitates platelet recovery without increasing leukemia recurrence in haploidentical hematopoietic stem cell transplantation: a randomized, controlled clinical study. Stem Cells Dev. 2011;20(10):1679‐1685. 21142788 10.1089/scd.2010.0447

[4] Ijaz A , Khan AY , Malik SU , et al. Significant risk of graft‐versus‐host disease with exposure to checkpoint inhibitors before and after allogeneic transplantation. Biol Blood Marrow Transplant. 2019;25(1):94‐99. 30195074 10.1016/j.bbmt.2018.08.028 PMC6310648

[5] Gress RE , Miller JS , Battiwalla M , et al. Proceedings from the National Cancer Institute's Second International Workshop on the Biology, Prevention, and Treatment of Relapse after Hematopoietic Stem Cell Transplantation: Part I. Biology of relapse after transplantation. Biol Blood Marrow Transplant. 2013;19(11):1537‐1545. 24018395 10.1016/j.bbmt.2013.08.010 PMC3922045

[6] Konopleva M , Benton CB , Thall PF , et al. Leukemia cell mobilization with G‐CSF plus plerixafor during busulfan–fludarabine conditioning for allogeneic stem cell transplantation. Bone Marrow Transplant. 2015;50(7):939‐946. 25867648 10.1038/bmt.2015.58 PMC4490031

[7] Duan C‐W , Shi J , Chen J , et al. Leukemia propagating cells rebuild an evolving niche in response to therapy. Cancer Cell. 2014;25(6):778‐793. 24937459 10.1016/j.ccr.2014.04.015

[8] Chiarini F , Lonetti A , Evangelisti C , et al. Advances in understanding the acute lymphoblastic leukemia bone marrow microenvironment: from biology to therapeutic targeting. Biochim Biophys Acta (BBA)‐Mol Cell Res. 2016;1863(3):449‐463. 10.1016/j.bbamcr.2015.08.015 26334291

[9] Reddy V , Iturraspe JA , Tzolas AC , Meier‐Kriesche H‐U , Schold J , Wingard JR . Low dendritic cell count after allogeneic hematopoietic stem cell transplantation predicts relapse, death, and acute graft‐versus‐host disease. Blood. 2004;103(11):4330‐4335. 14962904 10.1182/blood-2003-09-3325

[10] Ciomber A , Mitrus I , Fidyk W , et al. Immunological properties of bone marrow microenvironment 1 year after allogeneic hematopoietic stem cell transplantation. Exp Hematol. 2016;44(12):1172‐1180. 27524270 10.1016/j.exphem.2016.08.001

[11] Remberger M , Törlén J , Ringdén O , et al. Effect of total nucleated and CD34+ cell dose on outcome after allogeneic hematopoietic stem cell transplantation. Biol Blood Marrow Transplant. 2015;21(5):889‐893. 25662230 10.1016/j.bbmt.2015.01.025

[12] Le Texier L , Lineburg KE , MacDonald KP . Harnessing bone marrow resident regulatory T cells to improve allogeneic stem cell transplant outcomes. Int J Hematol. 2017;105(2):153‐161. 27943115 10.1007/s12185-016-2161-5

[13] Isidori A , Salvestrini V , Ciciarello M , et al. The role of the immunosuppressive microenvironment in acute myeloid leukemia development and treatment. Expert Rev Hematol. 2014;7(6):807‐818. 25227702 10.1586/17474086.2014.958464

[14] Willem C , Makanga DR , Guillaume T , et al. Impact of KIR/HLA incompatibilities on NK cell reconstitution and clinical outcome after T cell‐replete haploidentical hematopoietic stem cell transplantation with posttransplant cyclophosphamide. J Immunol. 2019;202(7):2141‐2152. 30787107 10.4049/jimmunol.1801489

[15] Minnie SA , Kuns RD , Gartlan KH , et al. Myeloma escape after stem cell transplantation is a consequence of T‐cell exhaustion and is prevented by TIGIT blockade. Blood. 2018;132(16):1675‐1688. 30154111 10.1182/blood-2018-01-825240

[16] Shindiapina P , Alinari L . Pembrolizumab and its role in relapsed/refractory classical Hodgkin's lymphoma: evidence to date and clinical utility. Ther Adv Hematol. 2018;9(4):89‐105. 29623180 10.1177/2040620718761777 PMC5881987

[17] Norde WJ , Maas F , Hobo W , et al. PD‐1/PD‐L1 interactions contribute to functional T‐cell impairment in patients who relapse with cancer after allogeneic stem cell transplantation. Cancer Res. 2011;71(15):5111‐5122. 21659460 10.1158/0008-5472.CAN-11-0108

[18] Hajifathali A , Parkhideh S , Kazemi MH , Chegeni R , Roshandel E , Gholizadeh M . Immune checkpoints in hematologic malignancies: what made the immune cells and clinicians exhausted! J Cell Physiol. 2020;235:9080‐9097. 32383178 10.1002/jcp.29769

[19] Riaz N , Havel JJ , Makarov V , et al. Tumor and microenvironment evolution during immunotherapy with nivolumab. Cell. 2017;171(4):934‐949. 29033130 10.1016/j.cell.2017.09.028 PMC5685550

[20] Yared JA , Hardy N , Singh Z , et al. Major clinical response to nivolumab in relapsed/refractory Hodgkin lymphoma after allogeneic stem cell transplantation. Bone Marrow Transplant. 2016;51(6):850‐852. 26828905 10.1038/bmt.2015.346

[21] Koestner W , Hapke M , Herbst J , et al. PD‐L1 blockade effectively restores strong graft‐versus‐leukemia effects without graft‐versus‐host disease after delayed adoptive transfer of T‐cell receptor gene‐engineered allogeneic CD8+ T cells. Blood. 2011;117(3):1030‐1041. 21063028 10.1182/blood-2010-04-283119 PMC3035065

[22] Schroeder T , Rautenberg C , Haas R , Germing U , Kobbe G . Hypomethylating agents for treatment and prevention of relapse after allogeneic blood stem cell transplantation. Int J Hematol. 2018;107(2):138‐150. 29143282 10.1007/s12185-017-2364-4

[23] Godfrey J , Bishop MR , Syed S , Hyjek E , Kline J . PD‐1 blockade induces remissions in relapsed classical Hodgkin lymphoma following allogeneic hematopoietic stem cell transplantation. J Immunother Cancer. 2017;5(1):11. 28239465 10.1186/s40425-017-0211-z PMC5319147

[24] Holderried T , Fraccaroli A , Schumacher M , et al. The role of checkpoint blockade after allogeneic stem cell transplantation in diseases other than Hodgkin's lymphoma. Bone Marrow Transplant. 2019;54(10):1662‐1667. 30833743 10.1038/s41409-019-0498-0

[25] Kasamon YL , de Claro RA , Wang Y , Shen YL , Farrell AT , Pazdur R . FDA approval summary: nivolumab for the treatment of relapsed or progressive classical Hodgkin lymphoma. Oncologist. 2017;22(5):585‐591. 28438889 10.1634/theoncologist.2017-0004 PMC5423515

[26] Aslan A , Aras T , Özdemir E . Successful treatment of relapsed/refractory Hodgkins lymphoma with nivolumab in a heavily pretreated patient with progressive disease after both autologous and allogeneic stem cell transplantation. Leuk Lymphoma. 2017;58(3):754‐755. 27687237 10.1080/10428194.2016.1213835

[27] Younes A , Santoro A , Shipp M , et al. Nivolumab for classical Hodgkin's lymphoma after failure of both autologous stem‐cell transplantation and brentuximab vedotin: a multicentre, multicohort, single‐arm phase 2 trial. Lancet Oncol. 2016;17(9):1283‐1294. 27451390 10.1016/S1470-2045(16)30167-X PMC5541855

[28] Herbaux C , Gauthier J , Brice P , et al. Efficacy and tolerability of nivolumab after allogeneic transplantation for relapsed Hodgkin lymphoma. Blood. 2017;129(18):2471‐2478. 28270452 10.1182/blood-2016-11-749556

[29] Manson G , Mear J‐B , Herbaux C , et al. Long‐term efficacy of anti‐PD1 therapy in Hodgkin lymphoma with and without allogenic stem cell transplantation. Eur J Cancer. 2019;115:47‐56. 31082693 10.1016/j.ejca.2019.04.006

[30] Martínez C , Carpio C , Heras I , et al. Potential survival benefit for patients receiving allogeneic hematopoietic stem cell transplantation after nivolumab therapy for relapse/refractory Hodgkin lymphoma: real‐life experience in Spain. Biol Blood Marrow Transplant. 2020;26:1534‐1542. 32068094 10.1016/j.bbmt.2020.02.003

[31] Sindel A , Taylor T , Chesney A , Clark W , Fowler AA III , Toor AA . Hematopoietic stem cell mobilization following PD‐1 blockade: cytokine release syndrome after transplantation managed with ascorbic acid. Eur J Haematol. 2019;103(2):134‐136. 31140644 10.1111/ejh.13248

[32] Merryman RW , Kim HT , Zinzani PL , et al. Safety and efficacy of allogeneic hematopoietic stem cell transplant after PD‐1 blockade in relapsed/refractory lymphoma. Blood. 2017;129(10):1380‐1388. 28073785 10.1182/blood-2016-09-738385 PMC5345733

[33] Kassa C , Reményi P , Sinkó J , Kállay K , Kertész G , Kriván G . Successful nivolumab therapy in an allogeneic stem cell transplant child with post‐transplant lymphoproliferative disorder. Pediatr Tranplant. 2018;22(8):e13302. 10.1111/petr.13302 30345623

[34] Ravandi F , Assi R , Daver N , et al. Idarubicin, cytarabine, and nivolumab in patients with newly diagnosed acute myeloid leukaemia or high‐risk myelodysplastic syndrome: a single‐arm, phase 2 study. Lancet Haematol. 2019;6(9):e480‐e488. 31400961 10.1016/S2352-3026(19)30114-0 PMC6778960

[35] Wang AY , Kline J , Stock W , et al. Unexpected toxicities when nivolumab was given as maintenance therapy following allogeneic stem cell transplantation. Biol Blood Marrow Transplant. 2020;26:1025‐1027. 32018063 10.1016/j.bbmt.2020.01.021

[36] Abbas F , El Kossi M , Shaheen IS , Sharma A , Halawa A . Post‐transplantation lymphoproliferative disorders: current concepts and future therapeutic approaches. World J Transplant. 2020;10(2):29‐46. 32226769 10.5500/wjt.v10.i2.29 PMC7093305

[37] Gunes AK , Demir I , Pehlivan M . Classical Hodgkin lymphoma‐like post‐transplant lymphoproliferative disease after allogeneic stem cell transplantation for primary myelofibrosis is successfully treated with nivolumab: a case report. J Oncol Pharm Pract. 2020;27:509‐512. 32762294 10.1177/1078155220946462

[38] Wada F , Kondo T , Nakamura M , et al. Successful treatment of Hodgkin lymphoma‐like EBV‐associated post‐transplant lymphoproliferative disorder following allogeneic hematopoietic stem cell transplantation with nivolumab. Ann Hematol. 2020;99(4):887‐889. 32052134 10.1007/s00277-020-03960-4

[39] Zhang T , Xie J , Arai S , et al. The efficacy and safety of anti‐PD‐1/PD‐L1 antibodies for treatment of advanced or refractory cancers: a meta‐analysis. Oncotarget. 2016;7(45):73068‐73079. 27683031 10.18632/oncotarget.12230 PMC5341964

[40] Ansell SM , Lesokhin AM , Borrello I , et al. PD‐1 blockade with nivolumab in relapsed or refractory Hodgkin's lymphoma. N Engl J Med. 2015;372(4):311‐319. 25482239 10.1056/NEJMoa1411087 PMC4348009

[41] Dada R , Zekri J . Back to life with checkpoint inhibitors in Hodgkin lymphoma. J Oncol Pharm Pract. 2018;24(2):139‐142. 28597713 10.1177/1078155216685166

[42] Albring JC , Inselmann S , Sauer T , et al. PD‐1 checkpoint blockade in patients with relapsed AML after allogeneic stem cell transplantation. Bone Marrow Transplant. 2017;52(2):317‐320. 27892950 10.1038/bmt.2016.274

[43] Falchi L , Sawas A , Deng C , et al. High rate of complete responses to immune checkpoint inhibitors in patients with relapsed or refractory Hodgkin lymphoma previously exposed to epigenetic therapy. J Hematol Oncol. 2016;9(1):132. 27899158 10.1186/s13045-016-0363-1 PMC5129196

[44] Shad AT , Huo JS , Darcy C , et al. Tolerance and effectiveness of nivolumab after pediatric T‐cell replete, haploidentical, bone marrow transplantation: a case report. Pediatr Blood Cancer. 2017;64(3):e26257. 10.1002/pbc.26257 27650634

[45] Klobuch S , Weber D , Holler B , Herr W , Holler E , Wolff D . Potential role of the PD‐1/PD‐L1 axis in the immune regulation of chronic GVHD. Oncol Res Treat. 2017;40(7−8):447‐450. 28683452 10.1159/000471768

[46] McDuffee E , Aue G , Cook L , et al. Tumor regression concomitant with steroid‐refractory GvHD highlights the pitfalls of PD‐1 blockade following allogeneic hematopoietic stem cell transplantation. Bone Marrow Transplant. 2017;52(5):759‐761. 28067871 10.1038/bmt.2016.346 PMC9069644

[47] Beköz H , Karadurmuş N , Paydaş S , et al. Nivolumab for relapsed or refractory Hodgkin lymphoma: real‐life experience. Ann Oncol. 2017;28(10):2496‐2502. 28961828 10.1093/annonc/mdx341

[48] Haverkos BM , Abbott D , Hamadani M , et al. PD‐1 blockade for relapsed lymphoma post‐allogeneic hematopoietic cell transplant: high response rate but frequent GVHD. Blood. J Am Soc Hematol. 2017;130(2):221‐228. 10.1182/blood-2017-01-761346 PMC5510790 28468799

[49] Jiménez‐Ubieto A , Rodriguez A , Martinez Sánchez P , et al. Fatal graft‐versus‐host disease after allogeneic stem cell transplantation in a patient recently exposed to nivolumab. J Oncol Pharm Pract. 2019;25(2):502‐506. 10.1177/1078155217743069 29207936

[50] Chan TS , Sim JP , Kwong Y‐L . Low‐dose nivolumab‐induced responses in acute lymphoblastic leukaemia relapse after allogeneic haematopoietic stem cell transplantation. Ann Hematol. 2017;96(9):1569‐1572. 28573313 10.1007/s00277-017-3033-7

[51] Covut F , Pinto R , Cooper BW , et al. Nivolumab before and after allogeneic hematopoietic cell transplantation. Bone Marrow Transplant. 2017;52(7):1054‐1056. 28346414 10.1038/bmt.2017.44

[52] Onizuka M , Kojima M , Matsui K , et al. Successful treatment with low‐dose nivolumab in refractory Hodgkin lymphoma after allogeneic stem cell transplantation. Int J Hematol. 2017;106(1):141‐145. 28097534 10.1007/s12185-017-2181-9

[53] El Cheikh J , Massoud R , Abudalle I , et al. Nivolumab salvage therapy before or after allogeneic stem cell transplantation in Hodgkin lymphoma. Bone Marrow Transplant. 2017;52(7):1074‐1077. 28394366 10.1038/bmt.2017.69

[54] Terziev D , Hutter B , Klink B , et al. Nivolumab maintenance after salvage autologous stem cell transplantation results in long‐term remission in multiple relapsed primary CNS lymphoma. Eur J Haematol. 2018;101(1):115‐118. 29624748 10.1111/ejh.13072

[55] Ansell SM , Minnema MC , Johnson P , et al. Nivolumab for relapsed/refractory diffuse large B‐cell lymphoma in patients ineligible for or having failed autologous transplantation: a single‐arm, phase II study. J Clin Oncol. 2019;37(6):481‐489. 30620669 10.1200/JCO.18.00766 PMC6528729

[56] de Forceville L , Deau‐Fischer B , Franchi P , et al. Radiotherapy in combination with nivolumab for relapsed/refractory classical Hodgkin lymphoma: about two cases. Cancer/Radiothérapie. 2019;23(3):232‐239. 10.1016/j.canrad.2018.12.005 31147173

[57] Charles J , Giovannini D , Terzi N , et al. Multi‐organ failure induced by nivolumab in the context of allo‐stem cell transplantation. Exp Hematol Oncol. 2019;8(1):1‐8. 30963019 10.1186/s40164-019-0132-2 PMC6437980

[58] Wong E , Davis J , Koldej R , Szer J , Grigg A , Ritchie D . Nivolumab induces dynamic alterations in CD8 T‐cell function and TIM‐3 expression when used to treat relapsed acute myeloid leukemia after allogeneic stem cell transplantation. Leuk Lymphoma. 2020;61(1):185‐188. 31389281 10.1080/10428194.2019.1648803

[59] Lepik KV , Mikhailova NB , Moiseev IS , et al. Nivolumab for the treatment of relapsed and refractory classical Hodgkin lymphoma after ASCT and in ASCT‐naïve patients. Leuk Lymphoma. 2019;60(9):2316‐2319. 30712423 10.1080/10428194.2019.1573368

[60] Ogasawara R , Hashimoto D , Sugita J , et al. Loss of nivolumab binding to T cell PD‐1 predicts relapse of Hodgkin lymphoma. Int J Hematol. 2020;111(3):475‐479. 31538325 10.1007/s12185-019-02737-4

[61] Kobayashi T , Guo Y‐m , Yamashita T , et al. Relationship between clinical course of nivolumab‐related myositis and immune status in a patient with Hodgkin's lymphoma after allogeneic hematopoietic stem cell transplantation. Int J Hematol. 2019;109(3):356‐360. 30604316 10.1007/s12185-018-02584-9

[62] Quéro L , Gilardin L , Fumagalli I , et al. Anti‐PD‐1 immunotherapy in combination with sequential involved‐site radiotherapy in heavily pretreated refractory Hodgkin lymphoma. Cancer/Radiothérapie. 2019;23(2):132‐137. 10.1016/j.canrad.2018.05.002 30733172

[63] Chan T , Hwang YY , Khong PL , et al. Low‐dose pembrolizumab and nivolumab were efficacious and safe in relapsed and refractory classical Hodgkin lymphoma: experience in a resource‐constrained setting. Hematol Oncol. 2020;38:726‐736. 32786092 10.1002/hon.2787

[64] Sim JP , Lie AK , Ng M‐Y , Kwong Y‐L . Durable remission of T cell lymphoblastic lymphoma relapsing after allogeneic haematopoietic stem cell transplantation with a single low dose of nivolumab. Ann Hematol. 2021;100(9):2399‐2440. 32318779 10.1007/s00277-020-04042-1

[65] Allen PB , Savas H , Evens AM , et al. Pembrolizumab followed by AVD in untreated early unfavorable and advanced‐stage classical Hodgkin lymphoma. Blood. 2021;137(10):1318‐1326. 32992341 10.1182/blood.2020007400 PMC7955404

[66] Maly J , Alinari L . Pembrolizumab in classical Hodgkin's lymphoma. Eur J Haematol. 2016;97(3):219‐227. 27147112 10.1111/ejh.12770 PMC4987237

[67] Armand P , Shipp MA , Ribrag V , et al. Programmed death‐1 blockade with pembrolizumab in patients with classical Hodgkin lymphoma after brentuximab vedotin failure. J Clin Oncol. 2016;34(31):3733‐3739. 27354476 10.1200/JCO.2016.67.3467 PMC5791838

[68] Villasboas JC , Ansell SM . Nivolumab for the treatment of classical Hodgkin lymphoma after failure of autologous stem cell transplant and brentuximab. Expert Rev Anticancer Ther. 2016;16(1):5‐12. 26577822 10.1586/14737140.2016.1121812

[69] Chan TS , Luk T‐H , Lau JS , Khong P‐L , Kwong Y‐L . Low‐dose pembrolizumab for relapsed/refractory Hodgkin lymphoma: high efficacy with minimal toxicity. Ann Hematol. 2017;96(4):647‐651. 28138786 10.1007/s00277-017-2931-z

[70] Singh AK , Porrata LF , Aljitawi O , et al. Fatal GvHD induced by PD‐1 inhibitor pembrolizumab in a patient with Hodgkin's lymphoma. Bone Marrow Transplant. 2016;51(9):1268‐1270. 27111048 10.1038/bmt.2016.111

[71] Samoa RA , Lee HS , Kil SH , Roep BO . Anti‐PD‐1 therapy‐associated type 1 diabetes in a pediatric patient with relapsed classical Hodgkin lymphoma. Diabetes Care. 2020;43(9):2293‐2295. 32616607 10.2337/dc20-0740 PMC7440902

[72] Minson A , Douglas G , Bilmon I , Grigg A . Low dose PD‐1 inhibition in relapsed refractory Hodgkin lymphoma after allogeneic stem cell transplant with concomitant active GVHD. Br J Haematol. 2019;184(5):840‐844. 29532918 10.1111/bjh.15186

[73] Chan TS , Khong P‐L , Kwong Y‐L . Pembrolizumab for relapsed anaplastic large cell lymphoma after allogeneic haematopoietic stem cell transplantation: efficacy and safety. Ann Hematol. 2016;95(11):1913‐1915. 27473193 10.1007/s00277-016-2764-1

[74] Son S , Lee SG , Kim WK , Ahn Y , Jung JM . Stage IV natural killer/T‐cell lymphoma with chronic active Epstein–Barr virus, treated with pembrolizumab and TCRαβ‐depleted haploidentical hematopoietic stem cell transplantation. Leuk Lymphoma. 2020;21:1‐4. 10.1080/10428194.2020.1757666 32352338

[75] Bryant AR , Perales M‐A , Tamari R , Peled JU , Giralt S . Severe pembrolizumab‐associated neutropenia after CD34+ selected allogeneic hematopoietic‐cell transplantation for multiple myeloma. Bone Marrow Transplant. 2018;53(8):1065‐1068. 29515248 10.1038/s41409-018-0142-4 PMC6984164

[76] D'Souza A , Hari P , Pasquini M , et al. A phase 2 study of pembrolizumab during lymphodepletion after autologous hematopoietic cell transplantation for multiple myeloma. Biol Blood Marrow Transplant. 2019;25(8):1492‐1497. 30959163 10.1016/j.bbmt.2019.04.005

[77] Hsiao CC , Yao M , Liu JH , Chen WL . Pembrolizumab induced acute corneal toxicity after allogeneic stem cell transplantation. Clin Experiment Ophthalmol. 2018;46(6):698‐700. 29280537 10.1111/ceo.13139

[78] Khaddour K , Musiek A , Cornelius LA , et al. Rapid and sustained response to immune checkpoint inhibition in cutaneous squamous cell carcinoma after allogenic hematopoietic cell transplant for sézary syndrome. J Immunother Cancer. 2019;7(1):1‐7. 31801591 10.1186/s40425-019-0801-z PMC6894240

[79] Hammrich J , Wittig S , Ernst T , Gruhn B . CTLA‐4 polymorphism rs231775: Influence on relapse and survival after allogeneic hematopoietic stem cell transplantation in childhood. Eur J Haematol. 2019;102(3):251‐255. 30465728 10.1111/ejh.13200

[80] Qin X‐Y , Wang Y , Li G‐X , et al. CTLA‐4 polymorphisms and haplotype correlate with survival in ALL after allogeneic stem cell transplantation from related HLA‐haplotype‐mismatched donor. J Transl Med. 2016;14(1):100. 27118383 10.1186/s12967-016-0864-2 PMC4847362

[81] Zhou J , Bashey A , Zhong R , et al. CTLA‐4 blockade following relapse of malignancy after allogeneic stem cell transplantation is associated with T cell activation but not with increased levels of T regulatory cells. Biol Blood Marrow Transplant. 2011;17(5):682‐692. 20713164 10.1016/j.bbmt.2010.08.005 PMC2992836

[82] Ito A , Kondo S , Tada K , Kitano S . Clinical development of immune checkpoint inhibitors. BioMed Res Int. 2015;2015:605478. 26161407 10.1155/2015/605478 PMC4486755

[83] Bashey A , Medina B , Corringham S , et al. CTLA4 blockade with ipilimumab to treat relapse of malignancy after allogeneic hematopoietic cell transplantation. Blood. 2009;113(7):1581‐1588. 18974373 10.1182/blood-2008-07-168468 PMC2644086

[84] Davids MS , Kim HT , Bachireddy P , et al. Ipilimumab for patients with relapse after allogeneic transplantation. N Engl J Med. 2016;375:143‐153. 27410923 10.1056/NEJMoa1601202 PMC5149459

[85] Khouri IF , Fernandez Curbelo I , Turturro F , et al. Ipilimumab plus lenalidomide after allogeneic and autologous stem cell transplantation for patients with lymphoid malignancies. Clin Cancer Res. 2018;24(5):1011‐1018. 29246938 10.1158/1078-0432.CCR-17-2777 PMC5844825

[86] Gros FX , Cazaubiel T , Forcade E , et al. Severe acute GvHD following administration of ipilimumab for early relapse of AML after haploidentical stem cell transplantation. Bone Marrow Transplant. 2017;52(7):1047‐1048. 28581465 10.1038/bmt.2017.78

[87] Goodman AM , Rust M , Costello C , Ball ED . A 60‐year‐old man with progressive anemia while receiving checkpoint blockade therapy for relapsed myelofibrosis. Oncology. 2017;31(2):122‐124. 28205192

[88] Hobo W , Norde WJ , Schaap N , et al. B and T lymphocyte attenuator mediates inhibition of tumor‐reactive CD8+ T cells in patients after allogeneic stem cell transplantation. J Immunol. 2012;189(1):39‐49. 22634623 10.4049/jimmunol.1102807

[89] Sakoda Y , Park J‐J , Zhao Y , et al. Dichotomous regulation of GVHD through bidirectional functions of the BTLA‐HVEM pathway. Blood. 2011;117(8):2506‐2514. 21220749 10.1182/blood-2010-08-301325 PMC3062413

[90] del Rio M‐L , Jones ND , Buhler L , et al. Selective blockade of Herpesvirus entry mediator–B and T lymphocyte attenuator pathway ameliorates acute graft‐versus‐host reaction. J Immunol. 2012;188(10):4885‐4896. 22490863 10.4049/jimmunol.1103698 PMC3925259

[91] Wang DY , Salem J‐E , Cohen JV , et al. Fatal toxic effects associated with immune checkpoint inhibitors: a systematic review and meta‐analysis. JAMA Oncol. 2018;4(12):1721‐1728. 30242316 10.1001/jamaoncol.2018.3923 PMC6440712

[92] Kaloyannidis P , Al Shaibani E , Mashhour M , et al. De novo *Psoriasis Vulgaris* diagnosed after nivolumab treatment for refractory Hodgkin's Lymphoma, completely resolved after autologous hematopoietic stem cell transplantation. Case Rep Hematol. 2018;2018:6215958. 30410803 10.1155/2018/6215958 PMC6206568

[93] Wang AY , Kline J , Stock W , et al. Unexpected toxicities when nivolumab was given as maintenance therapy following allogeneic stem cell transplantation: toxicities with maintenance nivolumab after allogeneic stem cell transplantation. Biol Blood Marrow Transplant. 2020;26:1025‐1027. 32018063 10.1016/j.bbmt.2020.01.021

[94] Oran B , Daver N . Check‐point inhibitors before and after allogeneic hematopoietic stem cell transplant: the double‐edge sword. Biol Blood Marrow Transplant. 2019;25(1):e1‐e2. 30500441 10.1016/j.bbmt.2018.11.026

[95] Ghasemi K , Parkhideh S , Kazemi MH , et al. The role of serum uric acid in the prediction of graft‐versus‐host disease in allogeneic hematopoietic stem cell transplantation. J Clin Lab Anal. 2020;34:e23271. 32118321 10.1002/jcla.23271 PMC7370721

